# Automated FES for Upper Limb Rehabilitation Following Stroke and Spinal Cord Injury

**DOI:** 10.1109/TNSRE.2018.2816238

**Published:** 2018-03-29

**Authors:** Edmund F. Hodkin, Yuming Lei, Jonathan Humby, Isabel S. Glover, Supriyo Choudhury, Hrishikesh Kumar, Monica A. Perez, Helen Rodgers, Andrew Jackson

**Affiliations:** 1 Institute of Neuroscience, Newcastle UniversityNewcastle upon TyneNE2 4HHU.K.; 2 Miami Project to Cure ParalysisUniversity of MiamiMiamiFL33136USA; 3 Institute of NeurosciencesKolkata700017India; 4 Stroke Research GroupInstitute of Neuroscience, Newcastle UniversityNewcastle upon TyneNE2 4HHU.K.; 5 Stroke Service, Newcastle Hospitals NHS Foundation TrustRoyal Victoria InfirmaryNewcastle Upon TyneNE1 4LPU.K.; 6 Stroke Service, Northumbria Healthcare NHS Foundation TrustNorth Tyneside General HospitalNorth ShieldsNE29 8NHU.K.

**Keywords:** Associative plasticity, closed-loop, functional electrical stimulation, rehabilitation, stroke, spinal cord injury

## Abstract

Neurorehabilitation aims to induce beneficial neural plasticity in order to restore function following injury to the nervous system. There is an increasing evidence that appropriately timed functional electrical stimulation (FES) can promote associative plasticity, but the dosage is critical for lasting functional benefits. Here, we present a novel approach to closed-loop control of muscle stimulation for the rehabilitation of reach-to-grasp movements following stroke and spinal cord injury (SCI). We developed a simple, low-cost device to deliver assistive stimulation contingent on users’ self-initiated movements. The device allows repeated practice with minimal input by a therapist, and is potentially suitable for home use. Pilot data demonstrate usability by people with upper limb weakness following SCI and stroke, and participant feedback was positive. Moreover, repeated training with the device over 1–2 weeks led to functional benefits on a general object manipulation assessment. Thus, automated FES delivered by this novel device may provide a promising and readily translatable therapy for upper limb rehabilitation for people with stroke and SCI.

## I. Introduction

It is estimated that spinal cord injury (SCI) affects over 378,000 individuals each year, and 6 million people are living with SCI world-wide [1]. Incomplete tetraplegia is the most common form of SCI and regaining hand and arm use is ranked as the highest priority amongst tetraplegics [2]. Similarly, it is estimated that globally there are 33 million stroke survivors [3], and that three quarters will initially report upper limb weakness [4], with 45% still having limited fine hand use after 18 months [5]. A 2014 Cochrane review stated that no high-quality evidence can be found for any current upper limb interventions following stroke [6]. There is a clear need for new approaches to upper limb rehabilitation following neurological injury.

Neurorehabilitation aims to restore function following neurological injury by inducing neural plasticity. There is increasing evidence that the dosage (i.e. frequency and intensity of rehabilitation sessions) is critical for plasticity [7], [8] and that at present the dosage received by patients is small compared to those tested in animal models [9], [10]. It is therefore prudent that new approaches to upper limb rehabilitation facilitate an increase in the amount of therapy received.

Functional electrical stimulation (FES) involves applying peripheral stimulation to nerves in order to activate muscles, thereby inducing useful movement of an impaired limb. A recent meta-analysis for stroke rehabilitation suggested that FES interventions improved activity compared with both no intervention and training alone [11].

It has been proposed that the beneficial effects of FES during rehabilitation arise in part from neuroplastic changes in motor circuits [12]–[14]. Hebb’s principle (“Cells that fire together wire together” [15], [16]) suggests that the pairing of cortical and peripheral activity could strengthen intact descending pathways, and subsequently lead to improved motor function that is sustained after a therapeutic intervention has been completed [14], [17]–[20]. If so, then the therapeutic benefit of FES may rely on its pairing with appropriate descending commands, either by eliciting such activity directly by stimulating the cortex using transcranial magnetic stimulation (TMS) [21], [22], or by using a brain machine interface (BMI) to infer volitional intent, for example, using electroencephalography (EEG) [23], [24]. Where residual movements are present, an alternative approach is to use electromyography (EMG) [25]–[27], or motion tracking [28]–[30].

Various research groups have reported promising results using such approaches [19], [23]–[30], but the challenge remains to translate these often complex protocols into simple user-friendly devices suitable for intensive use in a clinical setting or at home. Additionally, to become commercially viable, devices must demonstrate efficacy, be cost effective, and be suitable for a wide range of patients [31].

In this paper, we introduce a novel approach to closed-loop control of FES for the rehabilitation of reach-to-grasp movements following stroke and SCI. We present a simple, low-cost device that automatically delivers assistive FES concurrent with the users’ volitionally activated movements. The device was designed to encourage repeated practice and, following an initial assessment, not require significant intervention by a therapist or caregiver. Its small size and relative portability make it practical for clinical and home-use.

## II. Methods

### A. Task & Device

**Fig. 1.**
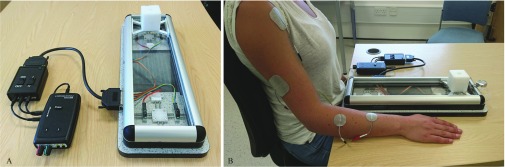
A – The automated FES device. Participants reached for the cube, grasped it and pulled it toward themselves along a rail of length 300 mm. When released, the cube automatically returned to the start position. Assistive stimulation was delivered by an Odstock Medical OS2CHS stimulator, modified to be controlled by a microcontroller (Arduino Micro) which received input from digital proximity sensors (Sharp GP2Y0D810Z0F) at either end of the rail. B – To stimulate wrist and finger extension the active electrode was positioned over extensor digitorum communis (EDC), and the indifferent electrode over extensor pollicis longus (EPL) and abductor pollicis longus (AbPL). To stimulate extension of the arm, the active electrode was placed over the anterior deltoid and the indifferent electrode over the triceps.

The device comprised of a custom-made slide rail, with integrated sensors and real-time link to a functional electrical stimulator via a microcontroller. As shown in Fig. 1, the device was placed on a flat surface in front of the participant, with the block at the far end of the rail. This was typically orthogonal to the table edge, but if necessary it was angled to aid reaching. A 5cm cube (60g) was fastened to the rail and tethered by a spring-loaded reel (max force approximately 2N) such that when displaced from start position and released, it automatically returned to the start position, ready for the next movement repetition. This allowed multiple cycles of the reaching and grasping task to be completed.

**Fig. 2.**
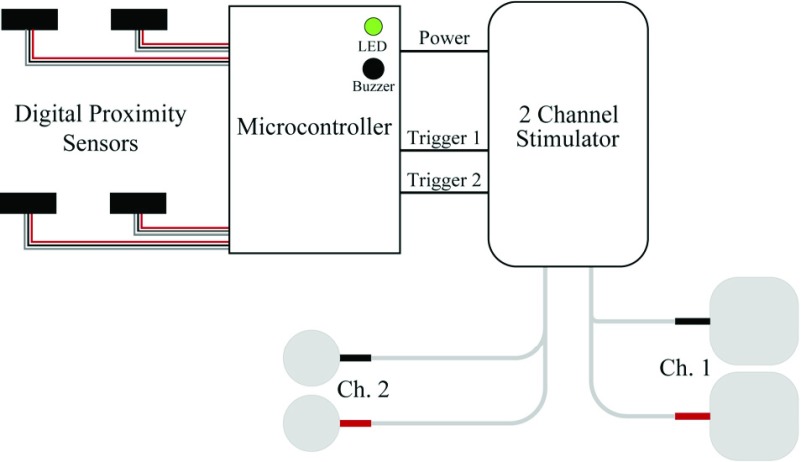
System schematic: The triggers for the two channel stimulator were controlled by a microcontroller. This received inputs from two sets of proximity sensors and used these signals to provide stimulation to open the hand and extend the arm at appropriate times during the reaching and grasping cycle.

**Fig. 3.**
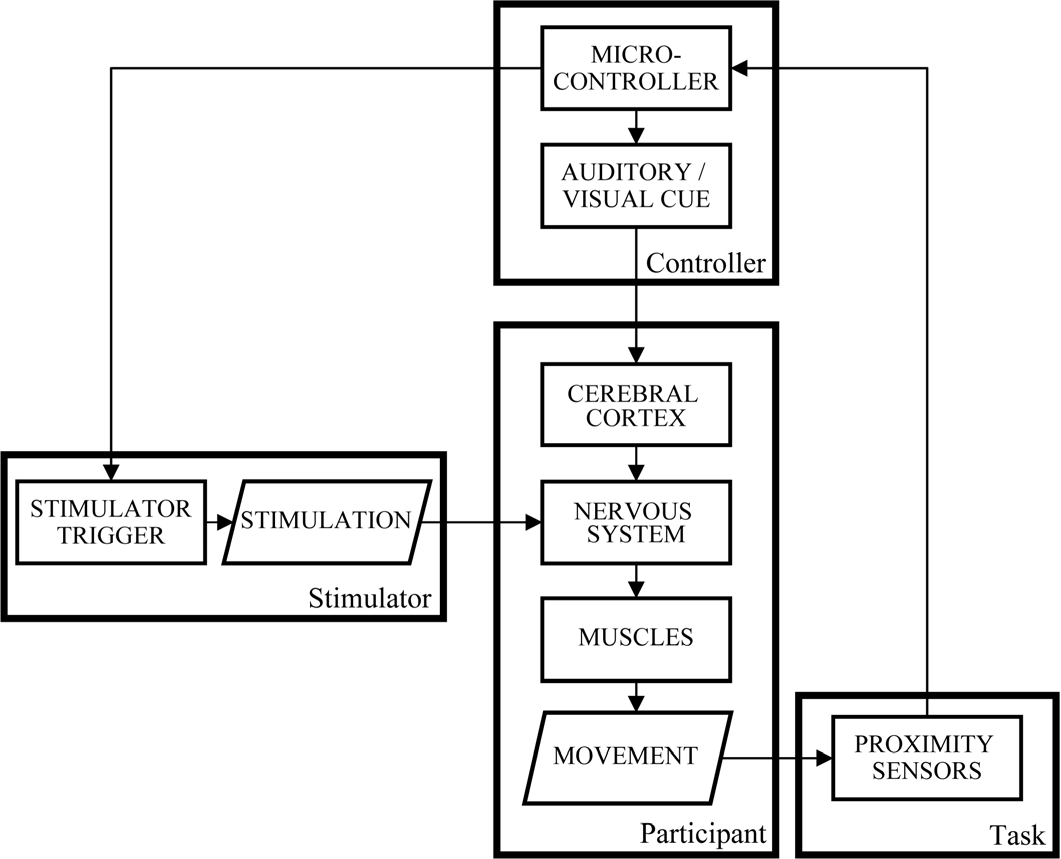
A diagram showing the closed-loop created by the device, stimulator, controller and participant in this study.

FES was delivered by a 2-channel stimulator (Odstock Medical Ltd OS2CHS) to open the hand and, for most participants, to extend the arm at the elbow. The trigger was modified to be controlled in real-time by a microcontroller (Arduino Micro) and digital proximity sensors with a 10cm range (Sharp GP2Y0D810Z0F) at either end of the rail. Auditory and visual cues (a short single (100ms) or double beep (2$\times$100ms) and LED illumination) were used to control task timing. Together with the sensors, this allowed the participant’s progress through each trial to be tracked so that stimulation of muscles could be delivered at the appropriate time, creating the closed-loop shown in Fig. 3.

At the start of each trial, auditory and visual cues indicated that the participant should reach towards and grasp the block. At the same time, stimulation was delivered to enhance this movement, e.g. stimulating the hand to open and the arm to extend. The end of the reaching phase was determined using a proximity sensor at the far-end of the slide to detect in real-time when hand was over the block. Thus stimulation was delivered through the whole outwards movement, irrespective of the movement duration. Once the block had been reached, stimulation was automatically turned off and participants pulled the block without assistance to the finish position. Again, proximity sensors were used to determine when the block had reached the finish position. Following a 1.5s delay, the participant received a further auditory and visual cue to release the block, and this was assisted with concurrent stimulation. Once released, the block returned automatically to the start position and triggered the end of stimulation. The next trial began after a rest period of 5s.

**Fig. 4.**
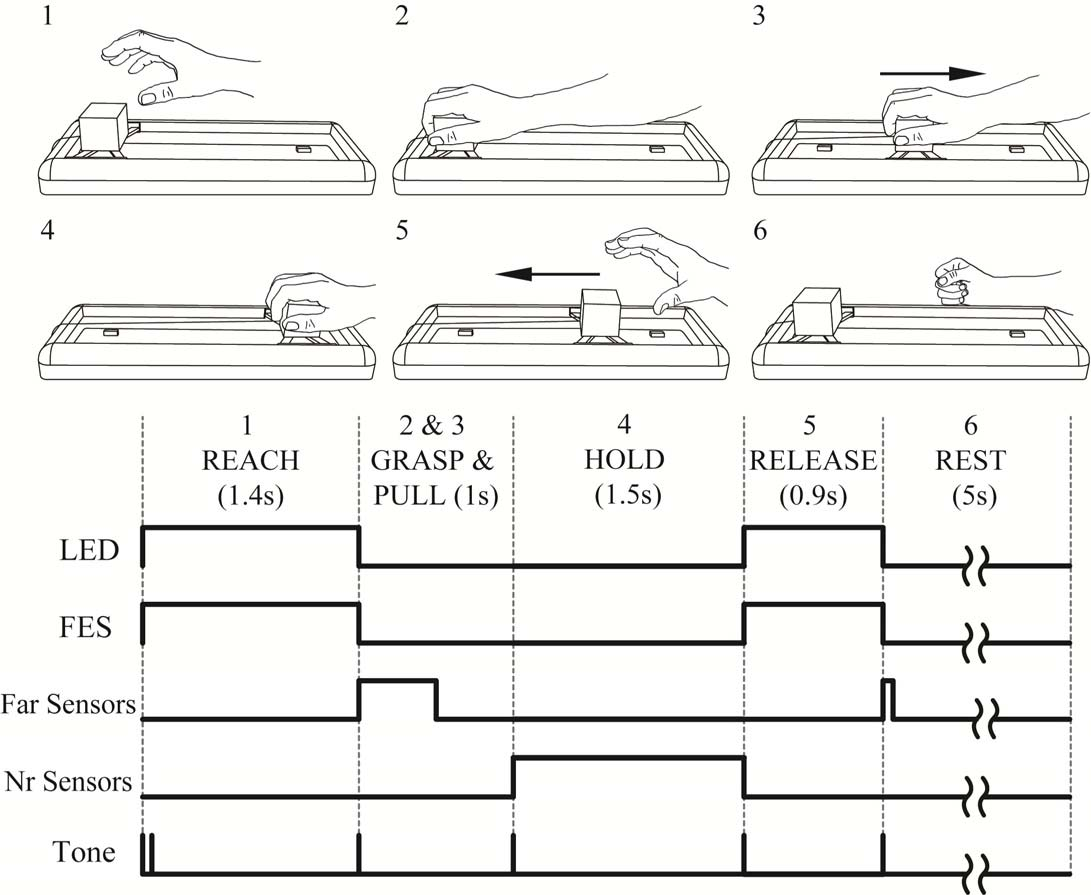
The intervention protocol: 1.The participant was given an auditory (double beep) and visual cue (LED on) to reach and grasp the 5cm cube, and FES was given to open the hand and, in most cases, extend the arm. 2. When proximity sensors (10cm range) detected that the open hand was over the block (marked by a single beep, LED off), the FES was turned off allowing the block to be gripped. 3. The participant pulled the block to the finish position with no FES assistance. 4. A proximity sensor detected the return was complete (single beep) and the microcontroller initiated a 1.5s delay. 5. Cues (single beep, LED on) indicated that the block should be released and FES was applied to open the hand. 6. When proximity sensors detected that the release was complete (the block was in the start position), FES was turned off (single beep, LED off). The participant then rested for 5 seconds before returning to step 1. Timings shown were calculated using data from participants with SCI (n = 7) for a block of 25 trials on day 3 of the intervention. Timings (mean (± SE)) are: Reach 1.4s (±0.2), Grasp and Pull 1.0s (±0.15), Hold 1.5s, Release 0.9s (±0.07), and Rest 5s. Similar timings were observed for participants with stroke.

The combination of cued movement initiation and automated detection of movement completion allowed stimulation to be reliably delivered contingent on the timing of the self-paced task epochs (e.g. reaching outwards and back) while maintaining a steady rate of progress through multiple trials. One purpose of this study was to determine whether this simple method of automated stimulation would complement the self-generated movements and be accepted by users. The protocol with further details of cues and timings is illustrated in Fig. 4.

### B. Pilot Study

Participants with chronic stroke and SCI ($\ge6$ months) were recruited to provide feedback on the device and complete a short intervention period. Four participants with stroke that met the inclusion criteria were recruited (mean age ± SE = 50 ±6 years, 4 male, mean time since stroke 6 ± 3 years, see Table I), one of whom was tested on two occasions 6 months apart. Seven participants with traumatic SCI were recruited (mean age ± SE = 37 ± 6 years, 6 male, mean time since SCI 8 ± 2 years, see Table II).

**TABLE I:** Participants for the Stroke Pilot Study

ID	Age	Gender	Side of Weakness	Left / Right Handed	Time since stroke onset (years)
1	57	M	Left	Right	5
2	67	M	Right	Right	16
3	40	M	Left	Right	2
4	37	M	Right	Right	2

**TABLE II:** Participants for the SCI Pilot Study

ID	Age	Gender	NLI^a^	ASIA^b^	SCIM^c^	Time since SCI (years)
1	20	M	C4	A	21	3
2	42	M	C4	C	30	9
3	20	M	C2	C	68	3
4	52	F	C5	C	99	14
5	57	M	C5	C	66	13
6	41	M	C7	C	64	4
7	29	M	C6	A	39	10

^a^ Neurological level of injury [32]

^b^ American Spinal Injury Association (ASIA) Impairment Scale [32]. ASIA A = complete, ASIA C = motor incomplete.

^c^ Spinal Cord Injury Measure (Version III, Self-report 2013) [33]

The study was completed at multiple sites: The Miami Project to Cure Paralysis (USA), Newcastle University (UK) and the Institute of Neurosciences, Kolkata (India). It was approved by the respective local ethics committees in all centres and all participants gave written informed consent prior to joining the study.

SCI participants attended 5 sessions and stroke participants 9 to 10, typically on consecutive days with breaks, such as weekends, as required. Sessions were scheduled to take 1 hour each, with a target of 200 repetitions per session. Three hours were scheduled for sessions at the start and end of the intervention to allow time to take consent, set-up the FES, perform assessments and to collect qualitative feedback. Participants aimed to complete blocks of 20 to 25 repetitions followed by 1 minute rest, although this was flexible to accommodate individual needs.

The inclusion criteria were that participants had chronic stroke or cervical SCI leading to mild, moderate or severe impairment of upper limb movement and an ARAT score less than 57 on the side to be trained. Participants were able to complete the task with FES assistance, aged over 18 years, and able to give informed consent. Participants were excluded as per the stimulator manufacturer guidelines (e.g. poorly controlled epilepsy, an implanted electronic device such as a pacemaker, or pregnancy).

Two of participants with SCI were categorised by the American Spinal Injury Association (ASIA) impairment scale as AISA A (complete injury) due to no sensory or motor function being preserved in the sacral segments S4-S5 [32]. However, they were able to elicit some voluntary force below the neurological level of injury, indicating residual connectivity. All other participants were categorised as ASIA C (motor incomplete).

Participants were assessed before and after the intervention period using the Action Research Arm Test (ARAT). Participants in the stroke pilot study were also assessed at 1 week and 1 month following the end of the intervention. ARAT is a reliable and validated measure of upper limb function [34], [35] that involves the assessment of grasp, grip, pinch and gross movements on a scale of 0 to 3. The maximum score per arm is 57 and both arms were tested. To avoid bias, blinded videos were evaluated by an independent assessor who was not involved in delivering the intervention following the study, this methodology has been established previously [36], [37].

Participants were also asked to complete a questionnaire to collect qualitative feedback about the intervention. In addition, a focus group of physiotherapists was set-up to collect qualitative feedback from a clinical perspective.

SCI participant questionnaires contained structured questions on upper-limb function such as the strength and the range of movement before and after the intervention. This was answered using a Likert scale. They were also asked if they would like to use the technology for rehabilitation, if they had benefited from the intervention and if they could use the technology independently. They were additionally provided with an unstructured section for general comments about the intervention.

The stroke participant questionnaire was a mix of structured (Likert scale) and unstructured questions about the stimulation, appropriateness of the task and other suggested improvements or feedback.

The questionnaire for the physiotherapist focus group comprised of predominately structured questions on upper limb rehabilitation, the use of FES, and feedback on the device following a demonstration. There was also the opportunity to provide additional unstructured comments.

### C. Stimulation

Asymmetric biphasic stimulation was applied using one or two pairs of disposable surface electrodes (PALS Neurostimulation Electrodes). The first pair ($\varnothing 3.2$cm round) extended the wrist, thumb and fingers, with the active electrode placed over the extensor digitorum communis (EDC), and the indifferent electrode over the extensor pollicis longus (EPL) and abductor pollicis longus (AbPL). Three individuals in the stroke study received this stimulation only. A second pair (5$\times$5cm square) was introduced later, and was used by all SCI participants and one stroke participant (Participant 2). This pair extended the arm at the elbow, with the active electrode on the anterior deltoid and the indifferent electrode on the triceps.

Stimulation parameters were individually set for each participant at the start of the study and checked for appropriateness before and throughout each session. Typically only slight adjustment was required during the intervention period. Current values ranged from 20 to 35mA and stimulation pulse widths of 130 to $350\mu \text{s}$ were used. The stimulation frequency was fixed at 40Hz, and electrodes were positioned on the first day, with the position marked using a UV pen. These electrode positions were maintained for the duration of the study with little adjustment required.

As the participants had some residual upper limb function, the intention was to enhance this rather than overpower it, thus ensuring participants were actively involved in the task. Electrode positions were based on the manufacturer’s guidelines [38] and adjusted to achieve the muscle activation that best resembled natural movement as observed by the experimenter and reported by the participant. The stimulation current was set at approximately 20mA and the pulse width increased until it produced a visible twitch in the index finger or arm. The pulse width was then increased to approximately 1.5 to 2.5 times this value as required to generate appropriate movement for the task. If this was not possible due to the maximum pulse width being reached, the current was increased and the process repeated.

Typically, in the absence of spasticity or muscle tightness, stimulation to the forearm would open the hand, including finger, wrist and thumb extension. Stimulation to the shoulder and triceps would extend the arm at the elbow, but only aid elevation from the table–elevation was predominately achieved by the participant’s residual function. In the presence of spasticity and muscle tightness, finger, thumb and elbow extension were reduced and some ‘clawing’ of the hand was observed. However, for the participants in this study, the stimulation delivered was sufficient to aid them in completing the task.

The proximity sensors, which were fitted on adjustable sliders, were positioned for each participant to allow for different hand sizes and reaching trajectories, which may otherwise lead to incorrect triggering of the sensors. After an initial training and setup period, it was uncommon for incorrect triggering to result in inappropriate stimulation.

## III. Results

### A. Task Compliance & Functional Outcomes - Stroke

All four participants completed the study, however the ARAT dataset for one participant (Participant 4) was incomplete and has not been shown here. The qualitative feedback from this participant is included in the analysis. Participants completed a total of 1800 to 2000 trials over the intervention period with each training session taking approximately 1 hour.

**Fig. 5.**
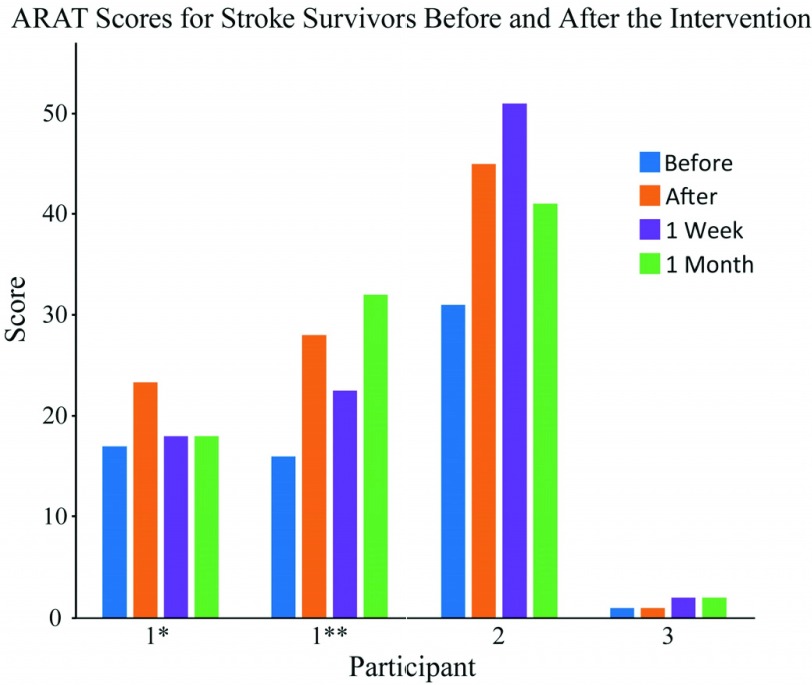
ARAT scores for stroke survivors completing the pilot study as assessed by the blinded, independent assessor. Assessments were completed before the intervention period, immediately after, and 1 week and 1 month after the completion of the intervention period. For reference, the original assessor’s scores for the before condition were: 10, 14, 29 and 3. Participant 4, who is not shown due to an incomplete dataset, had an original assessor score of 31. ^*^ indicates visit 1 and ^**^ indicates visit 2 for Participant 1, which were separated by 6 months.

Over the period of the intervention, ARAT scores improved by an average (± standard error) of 8 (± 3.1). Moreover, these improvements were maintained for 1 week (7 ± 4.5) and 1 month (7 ± 3.7) after the end of the intervention period. Two participants (1 and 2) achieved the minimal clinically important difference (MCID) for ARAT (set at 10% of the total score (${\ge}6$) [38]), as shown in Fig. 5. A clinically significant functional improvement was not found for the other participant. Note, however, that for this participant the ARAT may not have provided appropriate sensitivity as their score was at the extreme of the scale.

### B. Task Compliance & Functional Outcomes - SCI

SCI participants completed approximately 1000 repetitions over the 5 days. All participants completed the full period, and as planned, sessions (excluding assessments) took approximately 1 hour. The hand / side best suited to completing the task with FES assistance, as agreed with the participant, was trained during the intervention, with the untrained side acting as a control.

**Fig. 6.**
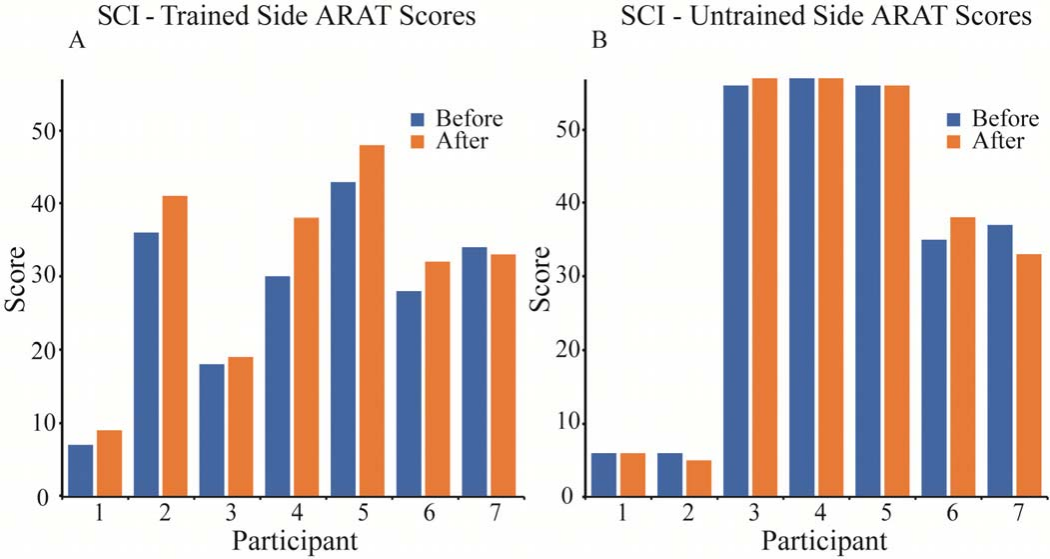
Panel A - The ARAT scores for the trained side for participants with SCI before and after the intervention. Panel B - The ARAT scores for the untrained side before and after the intervention. ARAT scores are as assessed by the blinded, independent assessor. For reference, the original assessor’s scores for the before condition for participants 1 to 7 were (trained / untrained): 8 / 7, 35 / 5, 16 / 55, 27 / 57, 41 / 56, 30 / 34 and 35 / 39 respectively.

**Fig. 7.**
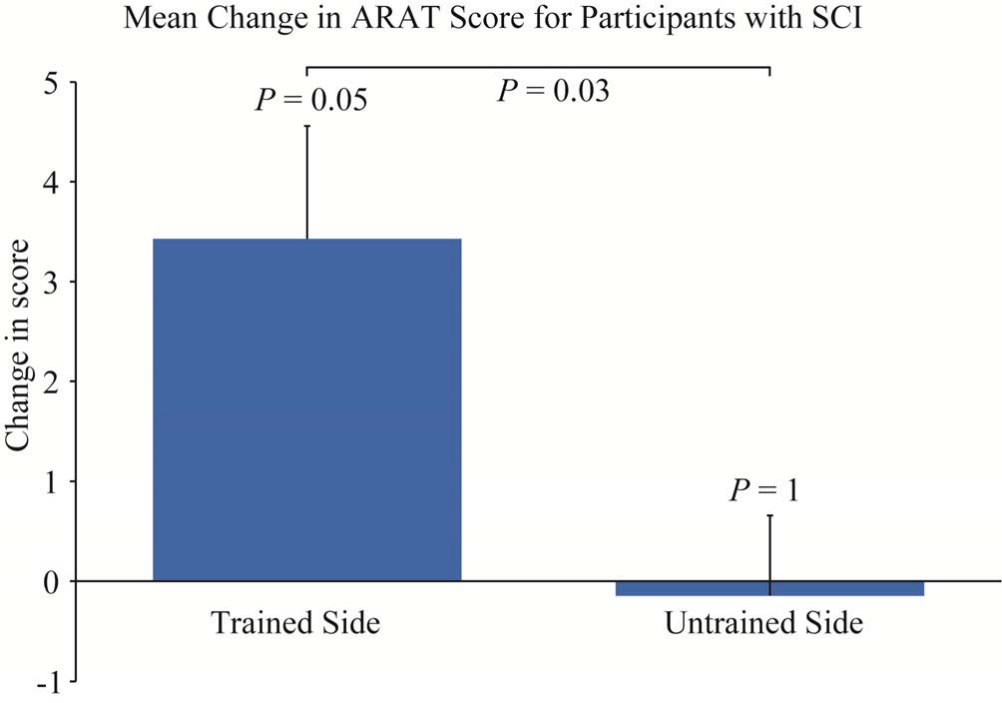
The mean change in ARAT score for the trained and untrained sides for participants with SCI. $P$ values show the statistical significance measured using the paired two-sided Wilcoxon signed-rank test for between the before and after conditions on the train and untrained sides (n = 7, ${T}^{+}= {26}. {5}$ and 5.5 respectively), and between the two sides (n = 7, ${T}^{+}= {21}$). Error bars show standard error.

ARAT scores were assessed before and after the intervention for both the trained and untrained limb (Fig. 6). The mean (± standard error) improvement in ARAT score was 3.4 (±1.1) on the trained side (Fig. 7). This was significantly greater than the change in the untrained side over the same period (0.1 ± 0.8, paired two-sided Wilcoxon’s signed-rank test, n = 7, $T^{+}=21$, P = 0.03). One SCI participant showed an improvement that exceeded the MCID (${\ge} 6$).

### C. Qualitative Feedback

All stroke participants reported that they would use the device again. Two participants (#1 and #2) noted in an unstructured question that they had experienced functional improvements such as better movement in the hand, being able to pick up objects and ability to complete bimanual tasks. All participants agreed that the stimulation was comfortable and that it helped them move their upper limb in a useful manner during the task. Two participants asked for the device to be smaller/more portable.

Six out of seven SCI participants reported that they had benefited from using the device, with 5 out of 7 saying that they would use it again. Three participants reported benefits with activities of daily living such as holding a pen, drinking and cutting food subsequent to using the device.

Nine physiotherapists with a range of experience from the National Health Service (NHS) in the North East of England attended the focus group. Seven agreed that the task and choice of muscles stimulated would be appropriate for a substantial proportion of stroke survivors they worked with, and if appropriate, 8 said that they would be happy to use the system. None of these therapists currently used FES more than ‘every once in a while’, with cost and availability of devices reported as barriers to use.

**Fig. 8.**
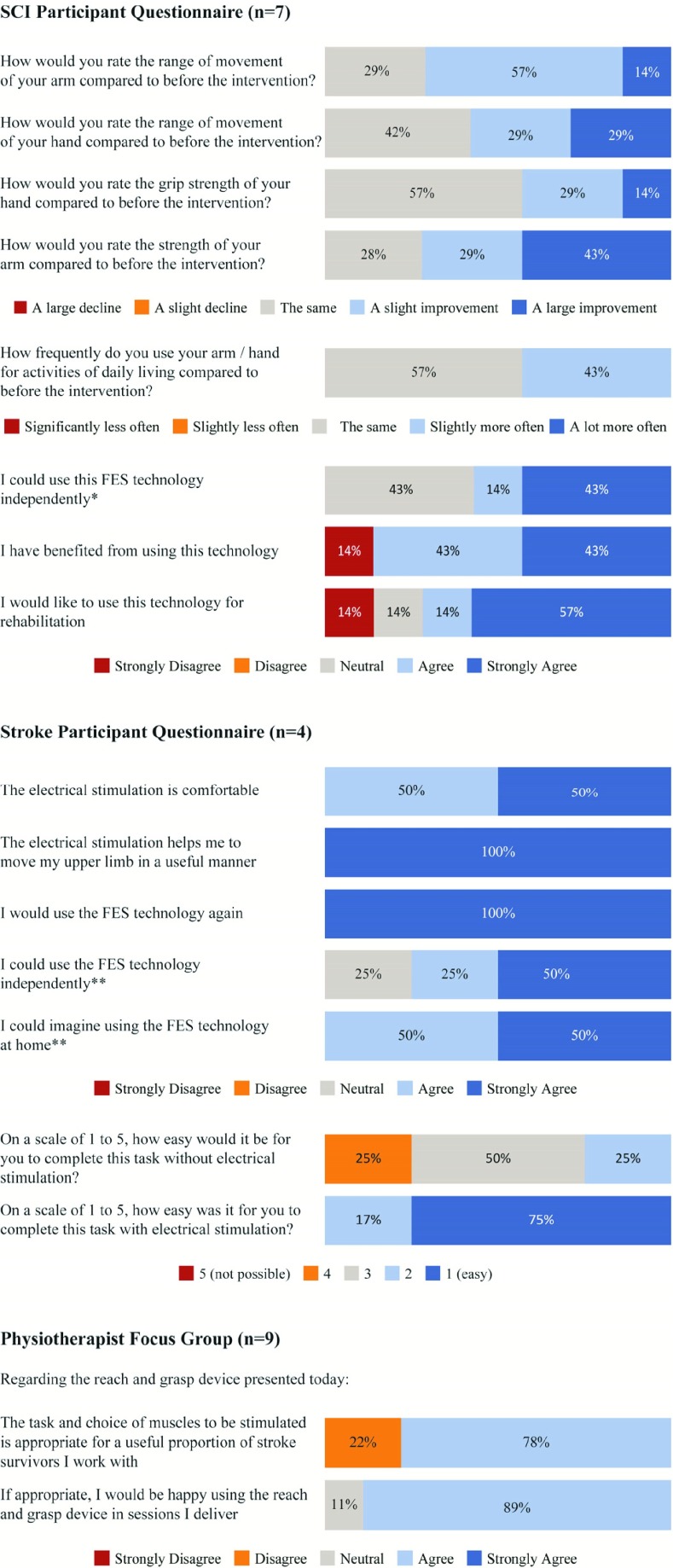
A selection of the qualitative data collected using a Likert scale. The number of respondents was 7, 4 and 9 for the SCI, stroke and physiotherapist groups respectively. ^*^ Participants stated that they would require assistance with initial set-up and placing of electrodes, but could otherwise use the device independently. ^**^ Participants often added the caveat that they would require training. Note that percentages may not add to 100% due to rounding error.

A selection of structured questions from across the 3 groups have been summarized in Fig. 8.

## IV. Discussion

We have presented a neurorehabilitation device for reach-to-grasp movements that is suitable for use by a subset of participants with SCI and stroke. The intervention was well-tolerated and produced measureable changes in a general upper limb function test after training for 1–2 weeks. Participants showed good compliance with the task and achieved the target number of repetitions. The majority of SCI participants reported that they had benefited from using the device and both groups typically agreed that they would use the device again. Feedback from the focus group demonstrated that if shown to be effective, the device was likely to receive a positive reception in a clinical environment.

Further studies will be required to establish whether additional benefits can be obtained through continued use of the device over extended time periods, and to assess whether these benefits are maintained. We speculate that the functional improvements we observed may be due to neuroplasticity arising from the temporal contingency of voluntary motor commands and peripheral stimulation, as well as exercise-dependent plasticity generated by completing a large number of repetitions of a task. However, additional investigations including neurophysiological testing and controls groups receiving FES or performing reaching movements alone will be required to support this hypothesis.

Improvement in ARAT scores amongst SCI participants were modest in comparison to the MCID ${\ge}6$ [39], with one participant (#4) showing an improvement greater than this clinically significant threshold. As final evaluations were completed immediately after the intervention on day 5, we cannot say how long-lasting effects were for the group. However, due to this participant’s improvement, they returned for a follow-up ARAT assessment one week after the intervention and it was found that the clinically significant benefit had been sustained.

It should be noted that in some instances the untrained hand had high levels of function, and this limits the comparability of the trained and untrained sides before and after the intervention.

Participants with stroke had additional follow-up sessions at 1 week and 1 month. Two participants (#1 and #2) showed a clinically significant increase in function, which appeared to be sustained for Participant 2. It is less clear for Participant 1, as he completed two intervention periods and appeared to lose the measured functional gains following the first intervention period, but sustain them following the second. However, he did retain some hand function following the first intervention as measured by the grasping subsection of the ARAT assessment (before 3/18, after 10/18, 1 week 7/18 and 1 month 8/18), but gains were offset by a drop in the scores in grip sub-section (before 7/12, after 8/12, 1 week 5/12, 1 month 4/12).

The grasping function was somewhat retained at the start of the second intervention and continued to progress (before 7/18, after 12/18, 1 week 14/18, 1 month 18/18), but gains were offset as the participant scored poorly in the grip subsection (before 0/12, after 7/12, 1 week 0/12, 1 month 7/12) in both the before and 1 week after assessments. This suggests that for this participant, the grip element of the ARAT may have been affected by other factors. While it is important not to draw strong conclusions from a single outcome measure for a small number of participants, there is some evidence for a carry-over effect, and the potential for activity dependent stimulation to lead to a carry-over effect has previously been reported [14], [19].

The two stroke participants (#1 and #2) that showed the clinically significant increase in function, initially scored in the mid-range of the ARAT. It could be inferred that participants with function within this range may benefit the most from using this device. Participant 3, who had a very low ARAT score, showed a very small change that was well below the MCID and may be attributed to many factors. A larger sample is required to understand the relationship between initial ARAT score and functional outcome.

Participants with residual sensory and motor function below the neurological level of SCI were included in this study. It was predicted that the largest changes in function would be seen in those classed as ASIA C (motor incomplete), as there should be greater residual connectivity. Indeed, as anticipated, participants who had complete SCI (#1 and #7) showed little to no improvement in ARAT score, although Participant 1 did verbally report feeling a benefit. Further studies will be required to establish optimal protocols for different severities of injury.

The reach and grasp movement can be broken down into three major components: (1) transporting the hand to the object, (2) the formation of the hand to grasp the object and (3) grasping the object [40]. One concern prior to the pilot study was whether this simple configuration of cues and proximity sensors would be sufficient to accurately facilitate this complex movement. Auditory and visual cues were delivered simultaneously with the beginning of stimulation, therefore not accounting for any reaction time, which may have varied across trials and participants. An alternative approach would be to trigger stimulation from the onset of movement, for example using brain signals [41], EMG [25]–[27], accelerometers or other motion tracking [28]–[30] to ensure precise timing between the descending motor command and peripheral stimulation. However, this increases the complexity and cost of such systems. In our study, participants reported the stimulation to be a help rather than a hindrance to task completion, suggesting that our simple automated closed-loop system was capable of delivering stimulation with timing that was appropriately coordinated with a participant’s intent. Further studies will be required to understand whether neurorehabilitative benefits can be improved by optimizing the timing of the stimulation train relative to motor intent.

It is important to note that this device does not allow the same level of flexibility as a physiotherapist led session. There is a trade-off between the low-cost and high repetitions provided by the device, and the personalized care provided by a therapist. However, the device does have in-built flexibility, the 5cm cube easily be swapped for an object of a different size, texture and shape, and the sensor positions adjusted accordingly. The distance reached can also be reduced, and there is the potential to upgrade the spring-loaded reel to include adjustable resistance. Stimulation parameters can be set to match the user’s needs, and the electrode positions adjusted to target specific muscles. Finally, it is conceivable that further devices based on similar principles of simple cueing and sensing of limb position could be developed for participants with higher or lower levels of function.

## V. Conclusion

This study has demonstrated a novel approach to closed-loop control of muscle stimulation for the rehabilitation of reach-to-grasp movements following stroke and SCI. Pilot data with a subset of people with upper limb weakness following SCI and stroke, has demonstrated usability of the device, with positive feedback from users, and modest functional benefits following a short intervention period. Further studies are required to establish clinical and cost effectiveness of longer durations of training, and to elucidate the mechanisms underlying functional improvements.
